# Extensive Horizontal Gene Transfer during *Staphylococcus aureus* Co-colonization In Vivo

**DOI:** 10.1093/gbe/evu214

**Published:** 2014-09-25

**Authors:** Alex J. McCarthy, Anette Loeffler, Adam A. Witney, Katherine A. Gould, David H. Lloyd, Jodi A. Lindsay

**Affiliations:** ^1^Institute for Infection and Immunity, St George’s, University of London, United Kingdom; ^2^Department of Clinical Sciences and Services, Royal Veterinary College, University of London, Hatfield, Hertfordshire, United Kingdom

**Keywords:** host adaptation, bacteriophage, plasmid, population dynamics, experimental evolution

## Abstract

*Staphylococcus aureus* is a commensal and major pathogen of humans and animals. Comparative genomics of *S. aureus* populations suggests that colonization of different host species is associated with carriage of mobile genetic elements (MGE), particularly bacteriophages and plasmids capable of encoding virulence, resistance, and immune evasion pathways. Antimicrobial-resistant *S. aureus* of livestock are a potential zoonotic threat to human health if they adapt to colonize humans efficiently. We utilized the technique of experimental evolution and co-colonized gnotobiotic piglets with both human- and pig-associated variants of the lineage clonal complex 398, and investigated growth and genetic changes over 16 days using whole genome sequencing. The human isolate survived co-colonization on piglets more efficiently than in vitro. During co-colonization, transfer of MGE from the pig to the human isolate was detected within 4 h. Extensive and repeated transfer of two bacteriophages and three plasmids resulted in colonization with isolates carrying a wide variety of mobilomes. Whole genome sequencing of progeny bacteria revealed no acquisition of core genome polymorphisms, highlighting the importance of MGE. *Staphylococcus aureus* bacteriophage recombination and integration into novel sites was detected experimentally for the first time. During colonization, clones coexisted and diversified rather than a single variant dominating. Unexpectedly, each piglet carried unique populations of bacterial variants, suggesting limited transmission of bacteria between piglets once colonized. Our data show that horizontal gene transfer occurs at very high frequency in vivo and significantly higher than that detectable in vitro.

## Introduction

*Staphylococcus aureus* is a commensal and opportunistic pathogen of humans and animals (DeLeo and Chambers 2009). Nasopharyngeal colonization can be considered as the first step toward the development of *S. aureus* infection*,* as most *S. aureus* infections are caused by endogenous colonizing strains (Von Eiff et al. 2001; McCarthy, Breathnach, et al. 2012). The emergence of methicillin-resistant *S. aureus* (MRSA) clones is a major global concern as options for prevention and treatment of infection are restricted. Human MRSA reservoirs have successfully evolved and adapted to survive as either healthcare or community pathogens. Livestock-associated (LA-)MRSA clones are an emerging problem, particularly in pig and veal calf farming, as these MRSA can successfully transmit from animal reservoirs and adapt to colonize and cause infections in in-contact humans (Huijsdens et al. 2006; Khanna et al. 2008; Wulf and Voss 2008; Smith et al. 2009; van Cleef, Monnet, et al. 2011). Although the pig-associated clonal complex (CC)398 lineage can cause human infection, they are currently inefficient at spreading between humans or within human hospitals (van Cleef, Graveland, et al. 2011; Wassenberg et al. 2011).

Populations of *S. aureus* isolates belong to many independently evolving lineages (also known as CCs), each with unique combinations of genes encoding surface proteins, secreted immune evasion proteins, and their regulators (Lindsay et al. 2006; McCarthy and Lindsay 2010, 2013). Mobile genetic elements (MGEs) account for 15–20% of the *S. aureus* genome (the mobilome) and include bacteriophages, plasmids, *S. aureus* pathogenicity islands (SaPIs), transposons, and the staphylococcal cassette chromosomes encoding methicillin-resistance (SCC*mec*) that can be acquired and lost from genomes through horizontal gene transfer (HGT) mechanisms (Lindsay 2014b). HGT of MGEs is assumed from comparative genomic studies to be a major evolutionary step that accelerates genetic and phenotypic variation in *S. aureus* populations, and enables adaptation to changing environments (Lindsay 2010). The remarkable variation in MGE content of *S. aureus* isolates within the same lineage, or even within the same MRSA clone, suggests that HGT is frequent among *S. aureus* isolates (Lindsay et al. 2006) although experimental evidence is lacking. As MGEs can carry genes encoding resistance, virulence, and/or host-specific factors that are relevant for survival and adaptation, their acquisition and loss is predicted to play important roles in adaptation to changing environments. Genome analysis suggests that particular MGEs have crossed-lineage boundaries and are associated with adaptation to specific environmental settings (Lindsay 2010). In particular, the φ3 family of bacteriophages encodes up to three human-specific host immune-evasion proteins, Sak*,* Chp, and Scn, and these bacteriophages are strongly associated with human rather than animal isolates (van Wamel et al. 2006; Sung et al. 2008; McCarthy, van Wamel, et al. 2012).

Polymorphisms in genes encoding proteins involved in specific host–pathogen interactions are also described in *S. aureus*; however, the distribution of polymorphism is often lineage-associated rather than host-associated (McCarthy and Lindsay 2010, 2013). Nevertheless, single nucleotide polymorphisms (SNPs) in the *S. aureus* genome might be expected to arise during host adaptation, particularly in immune evasion pathways or specific host binding proteins (Uhlemann et al. 2012). This type of gene polymorphism in specific pathways emerges in experimental evolution experiments utilizing pathogens such as *Pseudomonas aeruginosa* and *Escherichia coli* in response to new host environments (Ensminger 2013). In contrast, the potential for transfer of MGEs during bacterial host adaptation is poorly understood.

CC398 is the prevalent lineage of LA-MRSA colonizing pigs, veal calves, and in-contact humans in Europe and North America (McCarthy, van Wamel, et al. 2012). Although LA-MRSA can infect humans, it is inefficiently transmitted between humans or within human hospitals (van Cleef, Graveland, et al. 2011; Wassenberg et al. 2011). In contrast, a proportion of CC398 isolates appear to be associated with human-to-human transmission only, including infection outbreaks in the Netherlands, Belgium, Denmark and increasingly high levels of colonization reported in New York, Chicago, the Dominican Republic, and Dallas County Jail (van Belkum et al. 2008; Vandendriessche et al. 2011; Uhlemann et al. 2012; David et al. 2013). Pig-associated and human-specific *S. aureus* isolates of CC398 have substantial differences in their MGE content (McCarthy, van Wamel, et al. 2012). Pig-associated CC398 genomes typically carry SCC*mec* (β-lactam resistance), *tetM* (tetracycline resistance) on the transposon Tn*916*, φ2 and φ6 bacteriophages, SaPI5 with putative host specific proteins such as alternative *scn* variants (for complement binding) and *vwbp* variants (von willebrand factor binding and coagulation), and small plasmids such as those carrying genes encoding tetracycline (*tetK*) or trimethoprim resistance (*dfrG*) (Schijffelen et al. 2010; McCarthy, van Wamel, et al. 2012). The role of these MGEs in host adaptation has not been tested experimentally. On the other hand, the human-specific CC398 isolates, despite their geographical differences, typically carry φ3 bacteriophage (and genes encoding the immune evasion proteins Chp, Scn, and Sak) and alternative small plasmids (McCarthy, van Wamel, et al. 2012). These studies suggest that *S. aureus* mobilomes are genetically diverse and that individual mobilomes are well-adapted to colonization of specific hosts. However, the possibility that *S. aureus* mobilomes adapt to their hosts in short timescales has been little studied.

Experimental evolution is a powerful method to directly test models of host–pathogen interaction (Ensminger 2013) that has not previously been applied to *S. aureus*. In this study we aimed to investigate *S. aureus* population survival, adaptation and diversification during colonization with CC398 clones adapted to different hosts. Such studies require a tightly controlled environment and for this reason a gnotobiotic piglet model was used (Giotis et al. 2012). Molecular methods identified regions of the *S. aureus* genome that diversified in response to colonization of gnotobiotic piglets, and detected high frequency HGT of MGEs rather than SNPs. Co-colonization of piglets with human-specific and pig-associated CC398 isolates showed *S. aureus* clonal coexistence and genome diversification during colonization rather than fixation of a single successful variant. Accumulation of specific bacteriophages and a resistance plasmid suggested that they may play a role in adaptive evolution during colonization.

## Materials and Methods

### Strains

The two parent CC398 isolates used in the studies were pig-associated isolate S0385 and human-specific isolate H398. S0385 was isolated in 2006 from a blood culture of a patient with endocarditis who had contact with pig farms. It was the first CC398 isolate to be sequenced and completely annotated (Schijffelen et al. 2010) (GenBank NC_017333), and was kindly supplied by Henrik Hasman. H398, renamed from strain TB27855 for this study, was isolated in 1998 from blood culture during an outbreak of *S. aureus* disease unrelated to pig exposure (van Belkum et al. 2008) and was kindly supplied by Willem van Wamel. Previous comparative genomics indicates that S0385 belongs to the pig-associated CC398 clade, whereas H398 belongs to the human-specific CC398 clade (McCarthy, van Wamel, et al. 2012). S0385 is phenotypically resistant to oxacillin and tetracycline and forms gray colonies, whereas H398 is resistant to erythromycin and forms white colonies.

### Gnotobiotic Piglet Colonization

All animal experiments in this study were approved by the Royal Veterinary College Ethics and Welfare Committee and licensed by the UK Home Office (PPL 70/6975). Piglets were delivered by sterile caesarean section into and housed in a sterile plastic and stainless steel isolator at 30 °C (Giotis et al. 26). Each gnotobiotic piglet was inoculated atraumatically at 2 weeks of age on the sacrum, by gently rubbing a cotton swab dipped in overnight bacterial culture of both S0385 and H398 adjusted to 7 × 10^7^ colony forming units (CFU)/ml. At 4 h, 2, 4, 12, and 16 days, piglets were separately swabbed in both nostrils, behind one ear and on the sacrum. Swabs had been premoistened in sterile water before sampling, and were aseptically removed from the isolator, the tips snipped off into sterile saline and vortexed for 35 s to disperse the bacteria. Serial dilutions were plated on brain heart infusion agar (BHIA) with or without 20 µg/ml tetracycline in duplicate and incubated overnight at 37 °C. The mean colony count of the two plates was used to derive the log_10_ CFU/ml per swab. Twenty distinct colonies of each color type were picked per piglet for each time point from the BHIA plates without antibiotic, and stored in brain heart infusion broth (BHIB) with 20% glycerol at −20 °C.

### Laboratory Growth

A single colony was inoculated into 20 ml of BHIB and incubated overnight at 80 rpm and 37 °C. Cultures were diluted to OD_600_ 0.03 and 200 µl of each isolate was inoculated into 20 ml of fresh BHIB and incubated overnight at 80 rpm and 37 °C. Daily subculture, where 200 µl of overnight culture was diluted into 20 ml of fresh BHIB, was continued for 16 days. At 4 h, 2, 4, 12, and 16 days, cultures were sampled and serial dilutions plated on BHIA with or without 20 µg/ml tetracycline in duplicate and incubated overnight at 37 °C. Colony numbers were counted to calculate CFU/ml, and 20 well-isolated colonies of each color type were picked and stored in BHIB with 20% glycerol at −20 °C. Experiments were repeated four times.

### Genetic Analysis

DNA from stored progeny bacteria was extracted using the PureElute Bacterial Genomic DNA preparation kit (EdgeBiosystems, UK) following the standard protocol with the addition of lysostaphin (Lindsay et al. 2006). The presence of ten MGEs was detected using polymerase chain reaction (PCR) (supplementary table S1, Supplementary Material online), and products detected by separation on 1.5% agarose gels.

Both parents, 15 H398 progeny and 5 S0385 progeny were chosen for whole genome sequencing using the Ion Torrent Personal Genome Machine (Ion PGM) in conjunction with Ion Torrent workflow reagents (Life Technologies, Paisley, UK). For each sample, 100 ng of genomic DNA was fragmented by sonication using a BioRuptor UD-200 (Diagenode, Belgium) and the library prepared using the Ion Plus Fragment Library Kit according to the manufacturer’s instructions. Briefly, fragmented DNA was end-repaired, ligated to Ion-compatible adapters and nick-translated. To enable multiplexing of four samples on one sequencing chip, barcoded adaptors were used (Ion Xpress Barcode Adapters 1-16 Kit). Each library was size-selected using a 2% E-Gel SizeSelect Agarose Gel (Life Technologies) to produce a median fragment size of approximately 330 bp, and then quantified by quantitative PCR (Ion Library Quantitation Kit). Four barcoded libraries were pooled in equimolar amounts before being clonally amplified on Ion Sphere Particles (ISP) using the Ion OneTouch instrument. The template-positive ISPs were then enriched using the Ion OneTouch ES, before being sequenced on the Ion 316 chip using a 200-bp sequencing kit. Sequence coverage for each isolate is reported in supplementary table S4, Supplementary Material online.

Sequence quality was assessed using Fastqc (http://www.bioinformatics.babraham.ac.uk/projects/fastqc/). Sequence assembly was performed using MIRA v3.9.4 (Chevreaux et al. 1999) with default parameters for Ion Torrent data. Manual analysis and sequence inspection were performed using the Artemis and ACT genome visualization tools (Rutherford et al. 2000; Carver et al. 2005). SNP-based phylogenetic reconstruction was performed using a method similar to Harris et al. (2012). Briefly, reads were mapped to the ST398 reference genome (RefSeq: NC_017333) using TMAP v3.4.0 (Ion Torrent, USA) and alignments sorted with Picard v1.76 (http://picard.sourceforge.net/). Per base alignment statistics were generated with samtools mpileup (v0.1.18) (Li et al. 2009) and variants called using bcftools v0.1.17-dev. Variant sites were filtered based on the following criteria: Mapping quality above 30, site quality score above 30, at least four reads covering each site with at least two reads mapping to each strand but with maximum depth of coverage 200, at least 75% of reads supporting site (DP4), allelic frequency (AF1) of 1. Sites which failed these criteria in any strain were removed from the analysis. Phylogenetic reconstruction was performed by maximum-likelihood inference using RAxML v7.4.2 (Stamatakis 2006) with a general time reversible model of nucleotide substitution and a GAMMA model of rate heterogeneity, branch support values were determined using 1,000 bootstrap replicates. Sequence data have been deposited in the European Nucleotide Archive with study accession number ERP004215.

## Results

**Fig. 1.—:**
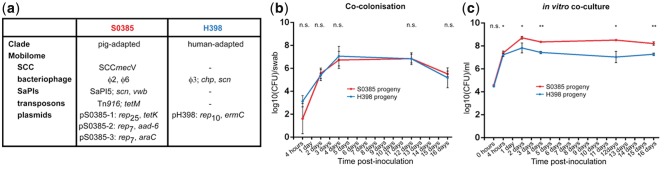
Coculture with S0385 and H398 isolates. (*a*) Origin and genetic features of parental *S. aureus* strains. (*b*) Growth of *S. aureus* strains during co-colonization of gnotobiotic piglets. (*c*) Growth of *S. aureus* strains during in vitro coculture in BHIB. Error bars represent one standard error of the mean. Comparison of bacterial growth was assessed using Student’s two-tailed *t*-test, *P < 0.05, **P < 0.01, ns, nonsignificant.

The two parental *S. aureus* CC398 isolates, pig-associated S0385 and human-specific H398, were sequenced and showed relatively conserved core genomes (supplementary fig. S1, Supplementary Material online), but differ substantially in their mobilomes (fig. 1*a*). S0385 (Schijffelen et al. 2010) carries the SCC*mecV* element and the Tn*916 tetM* transposon conferring methicillin and tetracycline resistance, respectively. In addition, S0385 carries 1) two bacteriophages, φ6 and φ2 which are highly similar but have distinctive integrase (*int*) genes and insert into unique specific locations on the chromosome, 2) one SaPI5 element with putative ruminant associated *scn* and *vwbp* variant genes implicated in complement evasion and coagulation, respectively, and 3) three small plasmids, one encoding *tetK* resistance, one with a putative *aad-6* gene for gentamicin resistance, and one with a putative regulator. In contrast, H398 carries the human associated φ3 bacteriophage encoding the human-specific *scn* and *chp* genes for complement evasion and neutrophil chemotaxis inhibition, respectively. H398 also carries a small plasmid with *ermC* erythromycin resistance. This MGE profile is typical of human-adapted CC398 (McCarthy, van Wamel, et al. 2012). Neither isolate carried *tra* genes necessary for conjugation. The surface gene *sdrE* was absent in S0385, as previously reported (Schijffelen et al. 2010), but was present in H398, and this difference was exploited by a specific PCR test (supplementary table S1, Supplementary Material online).

During gnotobiotic piglet co-colonization, both isolates grew and survived equally well (fig. 1*b*). As early as 4 h postinoculation of piglets, both isolates were colonizing all piglets at all sites sampled.

**Fig. 2.—:**
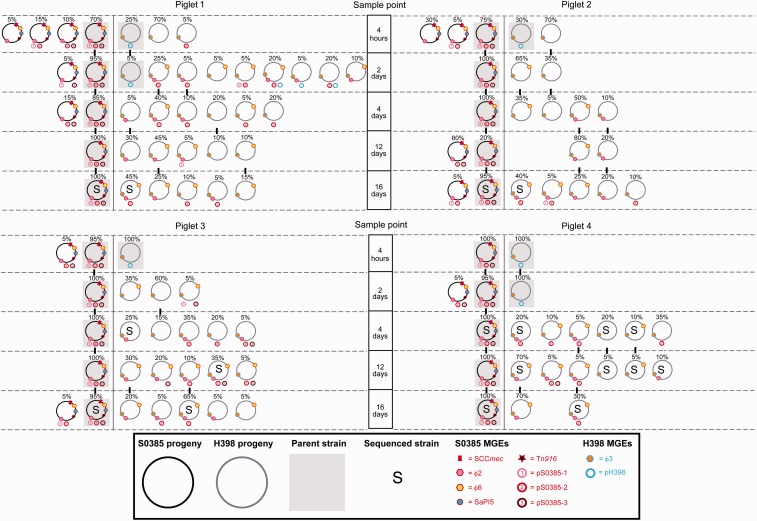
Highly diverse mobilomes of *S. aureus* progeny generated during co-colonization of gnotobiotic piglets. The unique mobilomes of 20 S0385 progeny (pig clade, black circle) and 20 H398 progeny (human clade, gray circle) for each piglet (shown as four different panels) at each sample point are shown. The frequency of each mobilome at a specific sample point is represented by a percentage above the mobilome. Individual MGEs have a colored border indicative of the parental strain they originate from, red border = originates from S0385, blue border = originates from H398. Parental mobilomes are represented by a gray shadow.

During piglet co-colonization, extensive acquisition and loss of MGEs was observed (fig. 2). In piglet 1 at 4 h, 30% of S0385 progeny had lost at least one plasmid (pS0395-1, pS0385-2, or pS0385-30), and 75% of H398 had lost plasmid pH398. Further, one H398 progeny had acquired a plasmid from S0385. By day 2, the genome of S0385 progeny had stabilized, but the H398 progeny genome had diversified into nine different mobilome types, each with different combinations of plasmids and bacteriophage acquired from S0385. By day 4, the parental H398 mobilome was not detected, and only two of the previous H398 progeny mobilomes were still present with four new mobilomes detected. Similarly at days 12 and 16, the S0385 genome with the original mobilome was stable, whereas the H398 progeny continued to show evidence of MGE acquisition and loss with new mobilomes emerging. Overall, a variety of mobilomes were detected in the H398 progeny over the 16-day co-colonization period rather than dominance of a single mobilome type. The S0398 elements SCC*mec*, Tn*916* and SaPI5, and H398 element φ3, were stable and did not show any evidence of HGT.

**Fig. 3.—:**
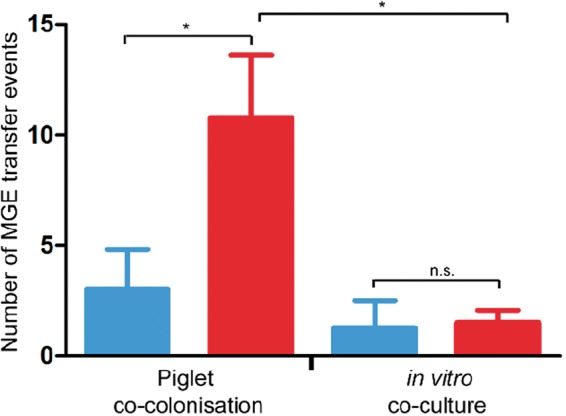
Minimum MGE transfer events (acquisition or loss) accounting for mobilome profiles detected in piglets and BHI. Significantly more HGT events were detected in the H398 background (red) in piglets than in the S0398 background (blue, *t*-test, *P* < 0.05). Significantly more HGT events were detected in the H398 background in piglets than in BHI (*t*-test, *P* < 0.01).

Piglets 2, 3, and 4 showed similar evidence of high frequency HGT of MGEs from S0385 to H398 as piglet 1 (fig. 2). The S0385 genome was stable in all progeny and in all piglets, and there was no evidence of any transfer of the H398 plasmid or ϕ3 bacteriophage to S0398. In contrast, in the H398 progeny the parental mobilome was replaced by new mobilomes that were detected at each time point by loss of the resident plasmid and frequent transfer of bacteriophage and plasmids from S0385. By day 4, on average each piglet had generated evidence of at least 7.5 transfer events into or out of the H398 parent, and by day 16 this had risen to 10.75 events (fig. 3).

Surprisingly, the H398 progeny between the individual piglets varied. For example, at 4 days, the dominant H398 mobilome in piglet 1 (φ2, φ3, φ6, pS0385-2) was not found in piglet 2 or 3 and accounted for only 5% of progeny in piglet 4. Similarly, the dominant mobilome in piglet 2 (φ2, φ3, φ6) was not found in piglet 1, 3, or 4; the dominant mobilome in piglet 3 (φ2, φ3, pS0385-2) was not found in piglet 2 or 4 and accounted for only 10% of progeny in piglet 1; and the dominant mobilome in piglet 4 (φ3, pS0385-2) was not found in piglet 2, and accounted for only 20% of progeny in piglets 1 and 3. This indicates relatively infrequent transmission of bacteria between the piglets despite their close contact. Further evidence for lack of transmission is the finding that 11 of the 21 (52%) novel mobilomes in the H398 background were detected in only one of the piglets. Different patterns of MGE acquisition in H398 over time were observed for each piglet, including piglet 2 which did not acquire any plasmids until day 16, and piglet 1 colonized with 11 different H398 mobilomes by day 2 compared with piglets 2–4 colonized with a total of four mobilomes.

**Fig. 4.—:**
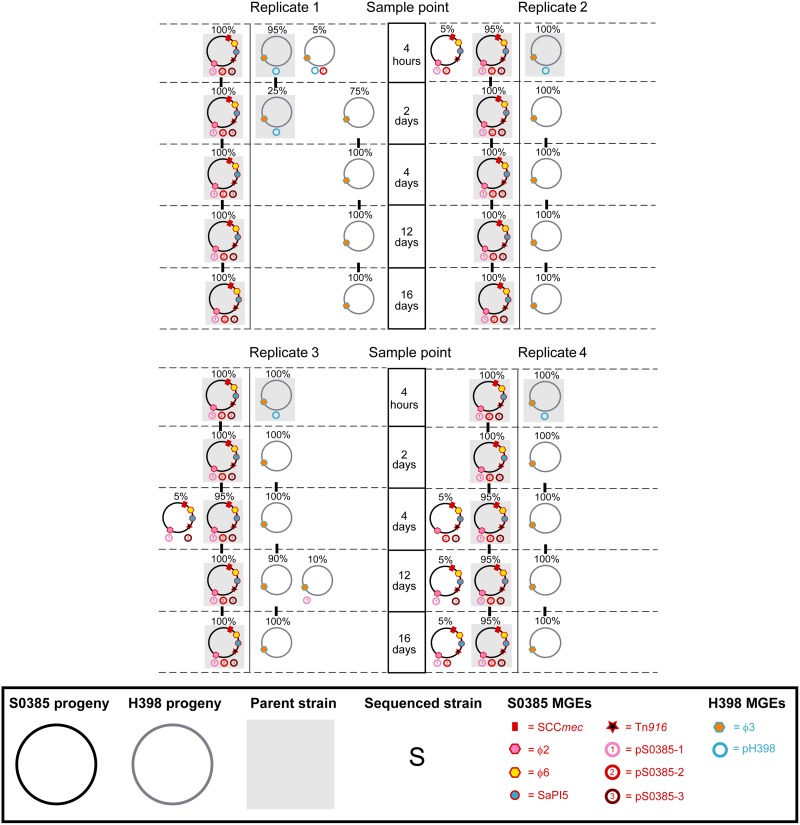
Stable mobilomes of *S. aureus* CC398 progeny generated during in vitro coculture in BHIB. The unique mobilomes of 20 S0385 progeny (pig clade, black circle) and 20 H398 progeny (human clade, gray circle) for each replication (shown as four different panels) at each sample point (4 h, 2, 4, 12, and 16 days postinoculation) are shown. The frequency of each mobilome at a specific sample point is represented by a percentage above the mobilome. Individual MGEs have a colored border indicative of the parental strain they originate from, red border = originates from S0385, blue border = originates from H398. Parental mobilomes are represented by a gray shadow.

We next investigated whether high levels of HGT occurred during co-colonization in vitro in rich laboratory media, ensuring that we used subcultures to capture sufficient generations and population size during the repeated cycles of growth in fresh media. During subculture of both isolates over 16 days in BHIB, the pig-associated isolate S0385 showed a clear fitness advantage over the human-specific isolate H398 by day 4 (fig. 1*c*). S0385 remained the dominant isolate for the remaining 16 days, although the H398 isolate did not disappear. These results are in contrast to the successful equal colonization of both isolates during the gnotobiotic piglet experiment. Genetic analysis of progeny bacteria from the laboratory media experiment (fig. 4) revealed less MGE movement than during colonization (fig. 3 and supplementary table S2, Supplementary Material online). The H398 progeny typically lost its plasmid pH398, and the S0385 progeny occasionally lost one plasmid (either pS0385-1, pS0385-2 or pS0385-3). On two occasions, a plasmid (pS0385-1 or pS0385-2) from S0385 was detected in a H398 progeny cell indicating HGT. Importantly, the majority of progeny retained the same mobilome as their parents.

Isolates marked in figure 2 with an S were chosen for whole genome sequencing. This confirmed that color of colony and *sdrE* PCR were reliable markers of each isolate’s background. Whole genome sequencing and variant site analysis identified 271 sites showing SNPs within the sequenced strain set. Phylogenetic reconstruction based on these sites confirmed separate clades corresponding to the two parental isolates (supplementary fig. S1, Supplementary Material online), with an estimated 268 SNPs between the two clades and one or less core genome SNPs per strain within the individual progeny clades (supplementary table S3, Supplementary Material online). There was no evidence of core genome SNP transfer between the isolates nor accumulation nor selection of SNPs. SNPs were not detected in genes encoding surface proteins or immune evasion proteins (supplementary table S3, Supplementary Material online). In contrast, substantial MGE movement was detected, correlating with the mobilome results (supplementary fig. S2, Supplementary Material online).

**Fig. 5.—:**
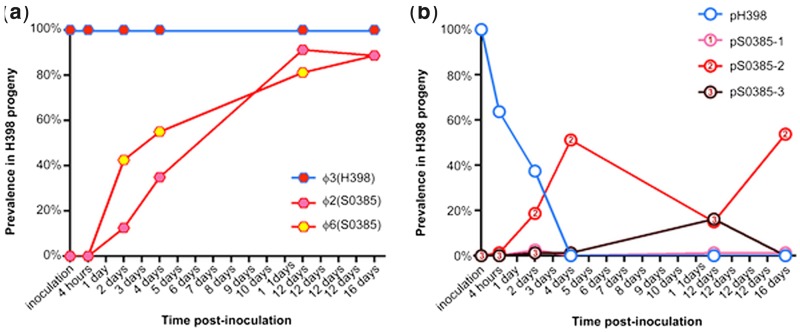
Incidence of bacteriophages (*a*) and plasmids (*b*) in H398 progeny during colonization of gnotobiotic piglets over time. A red border indicates an MGE that originated from S0385, and a blue border indicates an MGE that originated from H398. There was no evidence that SCC*mec*, SaPI5, or Tn*916* elements moved into the H398 progeny.

**Fig. 6.—:**
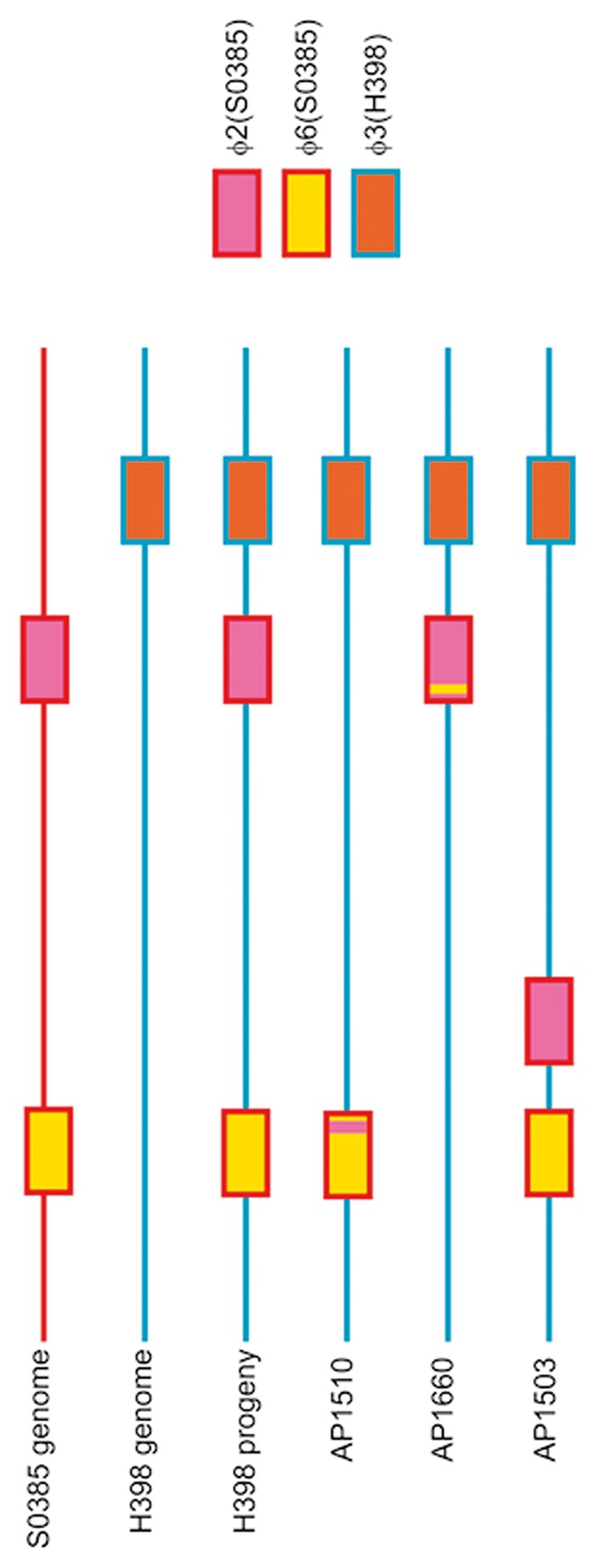
Bacteriophage acquisition and genome remodeling in H398 progeny. Parental genomes are represented by a single line (red = S0385 genome, blue = H398 genome) with the integration site of φ2, φ6, and φ3 bacteriophages shown. The majority of H398 progeny have the entire φ2 and φ6 bacteriophage integrated at known integration sites. AP1510 and AP1660 strains have unique φ2/φ6 recombinant bacteriophages integrated in their genomes. AP1503 has a φ2 bacteriophage integrated at a novel integration site.

During the course of piglet colonization experiments, φ2 and φ6 from S0385 accumulated in the H398 background (fig. 5). Not all progeny acquired both bacteriophages, but isolates with either or both bacteriophage were found at increased frequency over time. Sequencing analysis revealed the bacteriophages moved between the parents and progeny cleanly, although some variation was detected (fig. 6). Progeny AP1510 (piglet 3, day 4), in the H398 background, acquired φ6 but this bacteriophage had recombined with the φ2 resulting in an exchange of three genes (SAPIG1550–1552) for six genes (SAPIG0335–0341). This type of recombination event has not been detected during experimental culture before, and could result from recombination in the donor, or in the recipient followed by loss of the φ2. Progeny AP1660 (piglet 4, day 4) had a similar profile in reverse. This time, φ2 had been acquired, but it had recombined with φ6 in the same region resulting in the six genes (SAPIG0335–0341) for three genes exchange (SAPIG1550–1552). Genetic analysis also revealed evidence of bacteriophage integration into novel genomic sites (supplementary figs. S3 and S4, Supplementary Material online). Progeny AP1503 (piglet 3, day 4) had acquired a copy of the φ2 phage but it had integrated into a novel site; the 5′- and 3′-sequences of φ2 in AP1503 were both adjacent to SAPIG0656 (hypothetical protein). In the remaining φ2-positive H398 progeny (*n* = 11), φ2 had integrated into known integration site (SAPIG1555 in S0385), and φ6 had integrated into the known integration site (SAPIG0333 in S0385) in all φ6-positive H398 progeny (*n* = 12). This variation confirms that frequent and varied HGT and exchange of bacteriophages ϕ2 and ϕ6 from S0385 to H398 progeny, and that the amount of variation reported in figure 2 may be an underestimate. Furthermore, there was evidence of φ3 genes moving from H398 into AP2401, a progeny derived from S0385.

Plasmids also moved frequently during the course of the experiment (fig. 5). Although H398 rapidly lost its own plasmid (pH398), and occasionally acquired plasmids pS0385-1 and pS0385-3, it acquired and maintained plasmid pS0385-2. By 16 days, more than 50% of the H398 progeny harbored this plasmid. In contrast, other MGEs were stable throughout the experiment. All H398 progeny stably maintained the ϕ3 (fig. 5). The S0398 elements SCC*mec*, Tn*916*, and SaPI5 were stable and did not show any evidence of HGT. In addition, there was no evidence for the transfer of additional genomic regions between the isolates, including genomic islands and putative integrative and conjugative elements. Further, there was no evidence for the acquisition of MGEs that were not present in the parental strains.

## Discussion

Experimental evolution under controlled in vivo conditions in gnotobiotic piglets showed an unexpectedly high frequency of HGT between the co-colonizing *S. aureus* isolates. MGE transfer was detected within 4 h. Over subsequent days, an extensive range of variant mobilome profiles was continually generated. These data suggest that *S. aureus* populations can alter their genomes in vivo on much shorter timescales than previous estimates. Comparative genomic studies of human isolate variation during outbreaks and over longer term evolution of successful MRSA clones estimate that one SNP per genome is generated every 6 weeks (Harris et al. 2010; McAdam et al. 2012; Holden et al. 2013). MGE variation is often not investigated in these studies (Lindsay 2014a), but we have recently reported evidence of MGE movement as isolates spread between hospitalized patients (McCarthy, Breathnach, et al. 2012), and a recent study reported a “cloud” of variants colonizing a putative carrier in an outbreak of MRSA infection on a special care baby unit with minor variation in SNPs as well as the variable carriage of an erythromycin resistance plasmid (Harris et al. 2012). Our data are the first measurement of the transfer frequency of *S. aureus* MGE in vivo and suggest that it is capable of occurring at high frequency, and at significantly higher frequency than detected nucleotide polymorphisms in the genome.

HGT occurred at much higher frequency in the piglet colonization in vivo experiments than during growth in daily subculture of BHIB (fig. 3). The major mechanism of HGT in *S. aureus* is transduction through bacteriophage. In contrast, transformation is extremely inefficient in *S. aureus* (Morikawa et al. 2012; Lindsay 2014b). Although conjugation occurs in *S. aureus*, it is dependent on conjugative *tra* genes (McCarthy and Lindsay 2012) which are absent in the parent strains in this study. It is therefore likely that HGT was mediated by transduction (for the bacteriophage) and generalized transduction (for the plasmids), and the likely bacteriophages involved were φ2 and φ6. The triggers for generalized transduction and HGT in *S. aureus* are thought to be stress linked (Goerke et al. 2006; Selva et al. 2009). This suggests colonization in vivo or response to a new host induced transfer. Alternatively, there could be a trigger for successful MRSA clones to recognize appropriate conditions to enhance HGT when it is beneficial. Another possible explanation that we cannot rule out is that isolates with varied mobilomes were generated but did not survive in BHIB conditions.

A key finding of our study is that a diverse range of mobilomes was generated and survived during the colonization process, instead of the fixation of a single successful variant. Lieberman et al. (2014) have recently suggested that pathogens such as *Burkholderia dolosa* causing chronic long-term infection of cystic fibrosis patients evolve through SNP generation leading to the emergence of new “lineages” that continue to diversify, rather than fixation of specific SNPs and replacement with successful variants. Our study results suggest a similar evolutionary pattern in *S. aureus* during in vivo colonization, but through MGE transfer rather than SNPs.

A high frequency of transfer in vivo could explain the diversity of MGEs described in comparative population genomic studies of a wide variety of *S. aureus*, even between isolates belonging to the same lineage (Holden et al. 2004, 2013; Lindsay et al. 2006; Harris et al. 2010; McAdam et al. 2012; McCarthy, van Wamel, et al. 2012). We have also reported high levels of MGE and antibiotic resistance variation in hospital MRSA clones suggesting frequent movement into and out of isolates, possibly because resistances may have both a selective advantage and a fitness cost on the host bacterium in differing conditions (Knight et al. 2012, 2013; Zhou et al. 2013; Knoppel et al. 2014). Taken together, this data suggest that MGE transfer and loss is common and the high level detected in vivo suggests that sampling studies may be underestimating the variety of mobilomes in populations.

The variety of mobilomes detected during the colonization of the gnotobiotic piglets makes it difficult to conclude whether individual MGEs provided a selective advantage during colonization, or whether the variation is random and evolutionarily neutral (Zhou et al. 2013; Knoppel et al. 2014). Further experiments are required to establish whether any of the new mobilome types have a fitness advantage, compared with the parental strains, during piglet colonization. However, in this study systematic accumulation of both ϕ2 and ϕ6 bacteriophage was observed over time, and to a lesser extent accumulation of one plasmid (fig. 5). This suggests that selection may have occurred, and is consistent with a previous finding of ϕ2, ϕ6, and *rep*7 plasmid association with CC398 isolates from pig farming but not with isolates associated with human–human spread (McCarthy, van Wamel, et al. 2012). However, not all isolates in the gnotobiotic piglet study carried all three of these MGEs. The sequences of these MGEs do not clearly indicate genes that are expected to play a role in colonization (Schijffelen et al. 2010). The plasmid carries the aminoglycoside 6-adenylyltransferase (*aad-6*) gene; however, the piglets were not exposed to aminoglycosides nor any other antimicrobials during the experiment. It is also unclear why both bacteriophages accumulate in cells when their genomes are highly homologous. The bacteriophage may carry unknown selective advantage, such as host-specific factors that are not homologous to previously described genes implicated in host adaptation, or are only expressed during colonization (Koskiniemi et al. 2012). It is also possible that *S. aureus* HGT per se, driven by bacteriophages, provides a selective advantage during colonization. A pool of horizontally acquired genetic diversity in *S. aureus* populations may be a strategy for ensuring the raw material is available for efficient adaptability during the colonization of new hosts or to new niche environments (Knoppel et al. 2014).

HGT of MGEs during colonization only occurred in the direction of S0385 to H398. Transduction involves a proportion of the donor (S0385) population undergoing lysis and release of bacteriophage particles, and this may have only occurred in the S0385 background. Presumably this has a detrimental impact on S0385 fitness and survival. The HGT and growth data suggest the possibility of cooperation between the bacterial isolates rather than competition. An alternative hypothesis is that bacteriophages themselves control the lytic and transduction pathways, and as “selfish elements” ensure that they are spread throughout the bacterial population. *Staphylococcus aureus* bacteriophages have highly mosaic genomes, generated by frequent recombination, and each family integrates into unique but precise genomic locations (Kwan et al. 2005; McCarthy, Witney, et al. 2012). In this study, the generation of new bacteriophage by recombination between φ2 and φ6 bacteriophage, and integration of bacteriophage into unique sites of the H398 genome, was observed under in vivo experimental conditions. This is the first time to our knowledge that recombination has been seen under in vivo experimental conditions, and may be the result of bacteriophage coevolution (Sachs and Bull 2005). Collectively, the data in these studies provide evidence that new MGEs can be created during colonization with heterogeneous *S. aureus* populations. Of concern is the potential for new MGEs to emerge with new combinations of resistance, virulence, and/or host adaptation genes.

The high level of HGT between *S. aureus* isolates of the same lineage during piglet colonization suggests few genetic barriers to the transfer and successful replication and integration of certain MGEs. Recent research has revealed the genetic barriers in *S. aureus* that control transfer. Restriction–modification (R-M) systems have a critical role in ensuring that isolates of the same lineage exchange DNA at higher frequency than isolates of differing lineages (Waldron and Lindsay 2006; Roberts et al. 2013). This is the major barrier to HGT between *S. aureus,* which rarely carry CRISPR elements and rarely exchange DNA with other species (Lindsay 2014b). R-M systems likely explain why certain MGEs have not successfully transferred to all *S. aureus* lineages. A previous study of co-colonization of human volunteers with a human and a livestock *S. aureus* isolates belonging to different lineages (CC8 and CC398, respectively) saw the livestock isolate colonize more effectively than the human isolate in a subset of hosts. However, the two isolates did not belong to the same lineage, and no MGE exchange was detected (Slingerland et al. 2012). Therefore, it is likely R-M barriers prevented HGT, highlighting the importance of understanding the mechanisms that control HGT.

Unexpectedly, isolates with differing mobilomes did not appear to transmit between piglets despite very close physical contact in the isolation chamber. *Staphylococcus aureus* and MRSA are thought to spread between carriers by direct contact. After initial inoculation of the piglets, the *S. aureus* spread rapidly to all sampled piglet sites within 4 h. A likely explanation for this rapid dissemination is a lack of competing colonizing flora. By day 4, the maximum density of *S. aureus* on piglet skin was achieved, and transmission of isolates between piglets was low. The importance of colonizing flora in successful transmission of *S. aureus* between hosts is not well understood. Previous studies of piglets and humans have suggested that newborns are more readily colonized than older animals or humans (Peacock et al. 2003; Moodley et al. 2011), whereas pig farm workers typically become MRSA negative after as little as 24 h away from the farm and are therefore persistently positive for MRSA because of repeated exposure rather than colonization (van Cleef, Graveland, et al. 2011). The data here are consistent with the hypothesis that successful transmission of *S. aureus* and MRSA may be inefficient, especially if an established flora is already present. Gnotobiotic piglets may be a useful model for further investigation of *S. aureus* transmission and the importance of colonizing flora and antibiotic usage.

The high level of successful MGE transfer between CC398 clones during colonization suggests few barriers to the evolution of strains that can carry MGE that may allow them to colonize a variety of hosts, resist a wide range of antibiotics, and produce a diverse array of virulence factors. This is particularly concerning for the livestock industries, especially if LA-MRSA were to become carriers of the major food-poisoning toxins and to become more efficient at human–human transmission (European Food Safety Authority Panel on Biological Hazard 2009). Studies to establish whether particular mobilomes are better adapted to colonizing different hosts are needed, although preliminary studies growing these isolates in pig plasma or whole blood lead to extensive coagulation, indicating that this may be an unsuitable model for future studies.

Sampling of a single colony from colonized or infected hosts may not be representative of the diverse *S. aureus* populations harbored by an individual. This finding is potentially important for diagnostic laboratories reporting toxin or antibiotic susceptibility profiles if only a single colony is chosen for analysis. Interpretation of studies investigating epidemiological relationships between isolates during an outbreak or tracking the spread and evolution of successful clones may also be influenced by the choice of colony. Our results showed that the core genome is relatively stable compared with the MGEs, which is reassuring for transmission studies relying on core genome sequencing.

Measurement of MGE transfer between *S. aureus* revealed unexpectedly high frequencies during host colonization in vivo. The results in this study suggest that the coexistence of multiple mobilome types in vivo may be common, and that bacteriophage integrated into the genome plays a key role in colonization and gene transfer. These results have important implications for diagnostics, and for host–pathogen, epidemiological, evolutionary and transmission studies. Further in vivo experiments are required to determine exactly which MGEs and genes impact on fitness and host-adaptation.

## Supplementary Material

Supplementary figures S1–S4 and tables S1–S4 are available at *Genome Biology and Evolution* online (http://www.gbe.oxfordjournals.org/).

Supplementary Data
