# Supplementary material for: Extensive Horizontal Gene Transfer during Staphylococcus aureus Co-colonization In Vivo
Source: Genome Biol Evol. 2014 Sep 25;6(10):2697–708. doi: 10.1093/gbe/evu214 (PMC4224341; doi:10.1093/gbe/evu214)
Supplement: Supplementary Data [file supp_6_10_2697__index.html]

Extensive horizontal gene transfer during Staphylococcus aureus co-colonization in vivo — Extensive Horizontal Gene Transfer during Staphylococcus aureus Co-colonization In Vivo — Supplementary Data 

# Extensive Horizontal Gene Transfer during *Staphylococcus aureus* Co-colonization In Vivo

## Supplementary Data

files

**Files in this Data Supplement:**

- Supplementary Data - doc file
