# Supplementary material for: Extensive Horizontal Gene Transfer during Staphylococcus aureus Co-colonization In Vivo
Source: Genome Biol Evol. 2014 Sep 25;6(10):2697–708. doi: 10.1093/gbe/evu214 (PMC4224341; doi:10.1093/gbe/evu214)
Supplement: Supplementary Data [file supp_evu214_Suppl.doc]

**Supplementary Tables 1-4, Supplementary Figures 1-4.**

**Supplementary Table 1. PCR reactions and conditions.** All PCR reactions, except *mecA*, were performed using Bioline MyTaq kit (Bioline, UK) in 50 μl volumes containing 1pmol forward and reverse primer, 1 x buffer, 0.1 units Bioline MyTaq polymerase, 1ul of DNA and dH_2_O. Reactions were heated to 95 º C for an initial 2 minutes, followed by 35 cycles of 95 º C for 30 seconds, x ºC for 30 seconds and 72 ºC for 30 seconds, followed by of 1 cycle of 72 ºC for 10 minutes. *mecA* PCR was performed using Qiagen HotStarTaq (Qiagen, UK) in 50 μl volumes containing 1pmol forward and reverse primer, 1 x buffer, 0.1 units Qiagen HotStarTaq polymerase, 1mM dNTPs, 1ul of DNA and dH_2_O. Reactions were heated to 95 º C for an initial 5 minutes, followed by 35 cycles of 95 º C for 30 seconds, x ºC for 30 seconds and 72 ºC for 30 seconds, followed by of 1 cycle of 72 ºC for 10 minutes.

**Supplementary Table 2.** Minimum MGE events (acquisition or loss) detected in piglets, BHI and human plasma.

**Supplementary Table 3.** SNPs within CC398 clades from whole genome sequencing

**Supplementary Table 4.** Whole genome sequence coverage

**Supplementary Figure 1. Phylogenetic reconstruction of core genome SNP of parents and progeny confirms two clades.** Reference genome NC_017333, S0385 in triplicate and 5 S0385 progeny clustered separately to H398/TB2785 in triplicate and 15 H398 progeny. The scale bar represents mean number of substitutions per site.

**Supplementary Figure 2. Alignment of parent and progeny genomes.** The outer ring is the reference genome with reads in light blue. The next orange ring is the parent S0385, and the black rings are the S0385 progeny. Black dashes represent SNPs between S0385 and parent H398 (orange ring). The inner blue rings are 15 H398 progeny. The 6 and ɸ2 phage regions are highlighted in yellow.

**Supplementary Figure 3. Integration of ɸ2 and ɸ6 bacteriophage in H398 progeny genomes.** A) AP1510 was ɸ2 positive and ɸ6 negative. Alignment of contig 9 (upper) and contig 21 (lower) against the S0385 genome indicates that ɸ2 is inserted into the typical ɸ2 insertion site. Alignment of contig 84 against the S0385 genome confirms that no bacteriophage is inserted into the typical ɸ6 insertion site. B) AP1660 was ɸ2 negative and ɸ6 positive. Alignment of contig 4 against the S0385 genome confirms that no bacteriophage is inserted into the typical ɸ2 insertion site. Alignment of contig 24 (upper) and contig 46 (lower) against the S0385 genome indicates that ɸ6 is inserted into the typical ɸ6 insertion site. All figures produced using ACT. ɸ2 or ɸ6 in the S0385 genome are shown by red boxes, and ɸ2 and ɸ6 integrase (*int*) genes are also shown. The alignment of genome sequences/contigs are displayed in Artemis Comparison Tool. Blue/red bars present orthlogue matches identified by FASTA analysis.

**Supplementary Figure 4. Integration of ɸ2 at a novel insertion site in H398 progeny strain AP1503.** A) AP1503 contig 13 mapped across the typical ɸ2 insertion site but did not contain any ɸ2 gene sequences. AP1503 contig 49 contained the ɸ2 integrase (*int*) gene but the adjacent sequence was not homolgous to the typical ɸ2 insertion site. B) The 3’ and 5’ ends of the ɸ2 sequence mapped to AP1503 contigs 49 and 90, respectively. Both the downstream sequence of contig 90 and the upstream sequence of contig 49 mapped to the same region of the S0385 genome indicating that ɸ2 bacteriophage was integrated into a novel site (gene SAPIG0656) in the H398 progeny strain AP1503. All figures produced using ACT. ɸ2 sequences are shown by red boxes, ɸ2 integrase (*int*) gene, ɸ2 insertion site are also highlighted. The alignment of genome sequences/contigs are displayed in Artemis Comparison Tool. Blue/red bars present orthlogue matches identified by FASTA analysis.

Supplementary Table 1

| **MGE** | **Target Gene** | **Gene Identifier** | **Primer**  **Sequence (5’ to 3’)** | **Product size** | **Annealing temperature** |
| --- | --- | --- | --- | --- | --- |
| φ2 | *int* | SAPIG1554 | F: TTTGACTTTTCACGCGCTATTA  R: ACACCGGCGATTTGATTATTAC | 777bp | 60^o^C |
| φ3 | *int* | SAR2105 | F: TGAAAACACGTTGTTACGATGG  R: ATCCGCCTTCTTTGAAAATGTA | 780bp | 60^o^C |
| φ6 | *int* | SAPIG0334 | F: CCTTGAATTGATGGCGATTT  R: TTGCTGGGGCTGTAGAAGTT | 203bp | 60^o^C |
| SaPI5 | *int* | SAPIG0469 | F: ATTTTGCGAATTTATCAGCCA  R: CCGTTCCATATTGCCAAAAAG | 792bp | 60^o^C |
| SCC*mec* | *mecA* | SAPIG0042 | F: AAACCACCCAATTTGTCTGC  R: TCAGGTTACGGACAAGGTGA | 303bp | 60^o^C |
| Tn*916* | *tetM* | SAPIG0957 | F: ACTGCATTCCACTTCCCAAC  R: TGAAAATCCGCACCCTCTAC | 362bp | 64^o^C |
| pS0385-1 | *tetK* | PSAPIG010004 | F: ATCTGCTGCATTCCCTTCAC  R: GCAAACTCATTCCAGAAGCA | 818bp | 62^o^C |
| pS0385-2 | *aad*6 | PSAPIG020002 | F: ATTGCTCTCGAGGGTTCAAG  R: AAAATTCTCGTTCTGTTGGTTTTT | 387bp | 62^o^C |
| pS0385-3 | *araC* | PSAPIG030002 | F: AACTGCAACCCAAATTTCACT  R: TGGTTTACCCGAGCAACATT | 762bp | 61^o^C |
| pH398 | *rep*_10_ | pKH19_p1 | F: TGTTAGATATGATTGGCGGAA  R: TTGGTCGTCGCCTCTCATTA | 162bp | 60^o^C |
| sdrE | *sdrE* | SAR0567 | F: GGAGAGGTCATTGCTAAAGGA  R: GAATCACCATGAACCATTGGA | 236bp | 60^o^C |

The association between *sdrE* PCR positivity and colour on 100 colonies from mixed culture was significant (Chi^2^, *p* value <0.001).

Supplementary Table 2

|  | Piglet | | BHI | | Human plasma | |
| --- | --- | --- | --- | --- | --- | --- |
| Replicate | S0385 | H398 | S0385 | H398 | S0385 | H398 |
| 1 | 5 | 13 | 0 | 2 | 1 | 1 |
| 2 | 4 | 7 | 1 | 1 | 2 | 1 |
| 3 | 2 | 13 | 1 | 2 | 2 | 4 |
| 4 | 1 | 10 | 3 | 1 | 0 | 0 |
| Mean | 3 | 10.75 | 1.25 | 1.5 | 1.25 | 1.5 |

Supplementary Table 3

| Clade | Progeny strain | Nucleotide | SNP | Amino acid | Gene | Predicted gene function |
| --- | --- | --- | --- | --- | --- | --- |
| H398 | AP2304 | 1107721 | C-T | V-I | SAPIG1056 | *qoxC*, cytochrome aa3 quinol oxidase, subunit III |
| H398 | All (not parent) | 1146000 | C-T | H-Y | SAPIG1092 | dihydrolipoyllysine-residue acetyltransferase component of pyruvatedehydrogenase complex |
| H398 | AP2751 | 1416770 | C-T | R-R | SAPIG1350 | *opuT*, glycine betaine transporter |
| S0385 | AP2710 | 1021791 | C-A | G-G | SAPIG0973 | *oatA*, O-acetyltransferase (lipid metabolism) |

Supplementary Table 4.

| Isolate | Coverage |
| --- | --- |
| AP1503 | 41.6 |
| AP1510 | 42.6 |
| AP1654 | 43.4 |
| AP1660 | 41.5 |
| AP1753 | 46.4 |
| AP1772 | 42.7 |
| AP2153 | 27.3 |
| AP2301 | 27.8 |
| AP2304 | 32.4 |
| AP2310 | 30.7 |
| AP2321 | 57.8 |
| AP2328 | 34.6 |
| AP2401 | 49.3 |
| AP2421 | 48.1 |
| AP2551 | 46.6 |
| AP2603 | 37.5 |
| AP2710 | 37.5 |
| AP2751 | 33.8 |
| AP2882 | 38.2 |
| AP2971 | 37.8 |
| SO385 replicate 1 | 17.7 |
| SO385 replicate 2 | 16.1 |
| SO385 | 33.7 |
| H398 (TB27855) replicate 1 | 40.0 |
| H398 (TB27855) replicate 2 | 35.9 |
| H398 (TB27855) | 76.0 |

Supplementary Figure 1


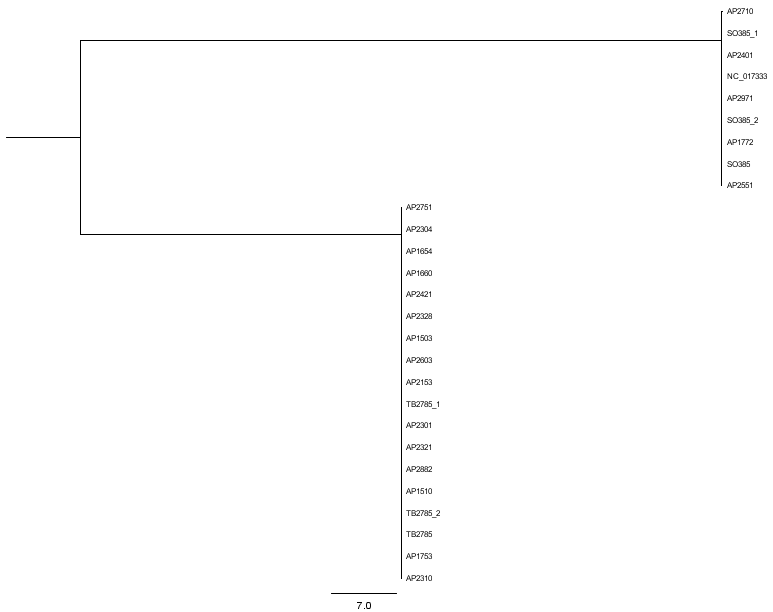


Supplementary Figure 2


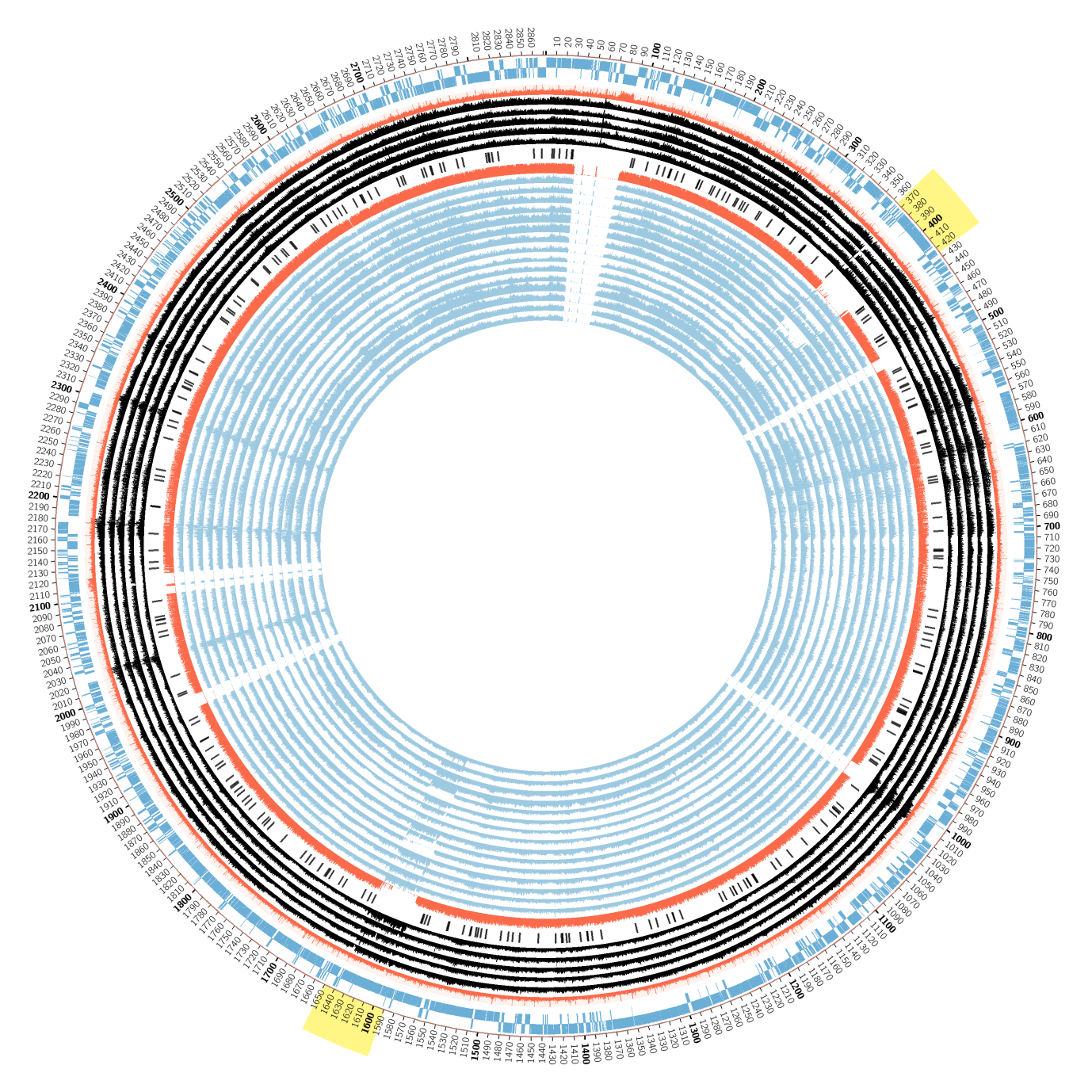


Supplementary Figure 3


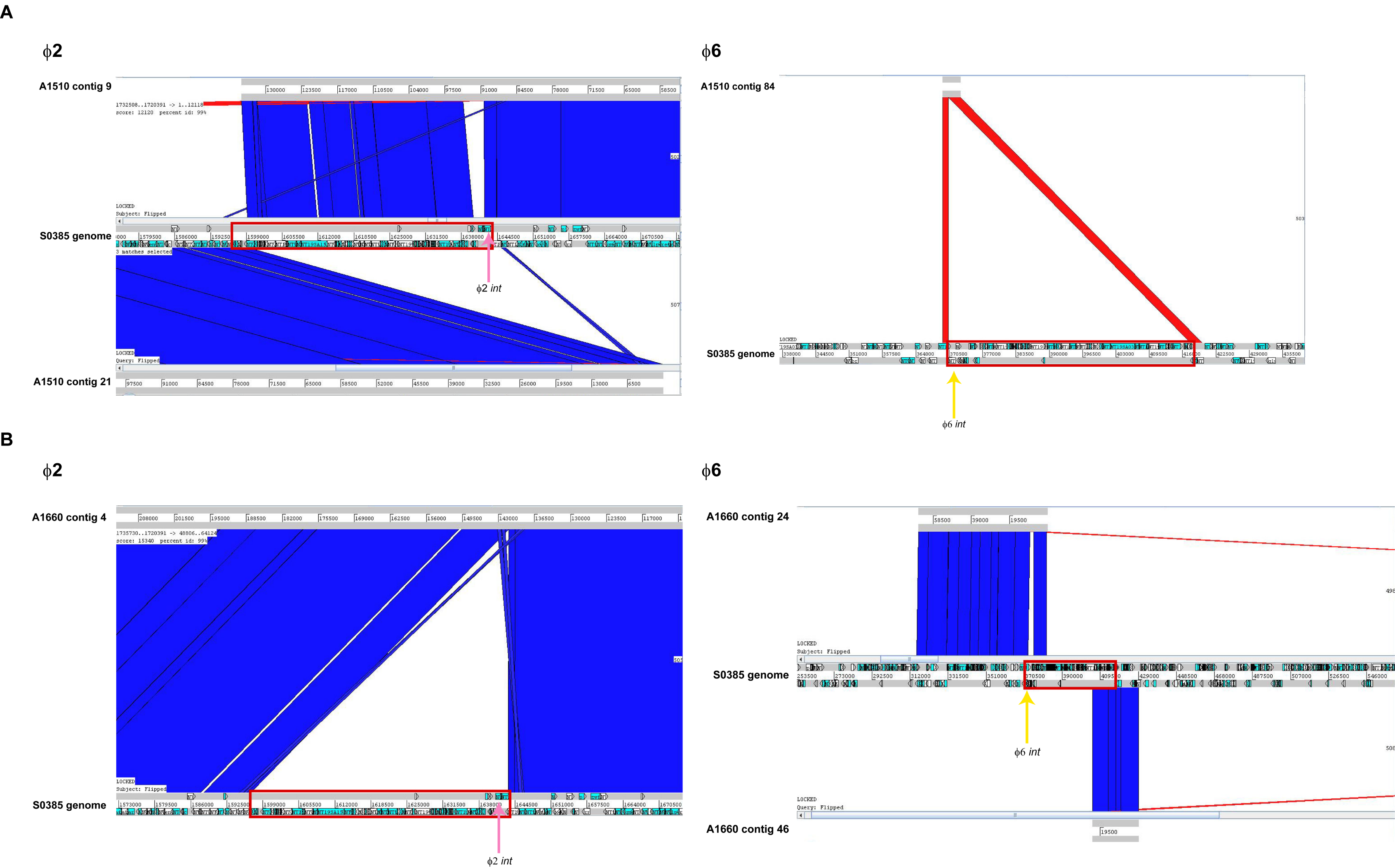


Supplementary Figure 4

**
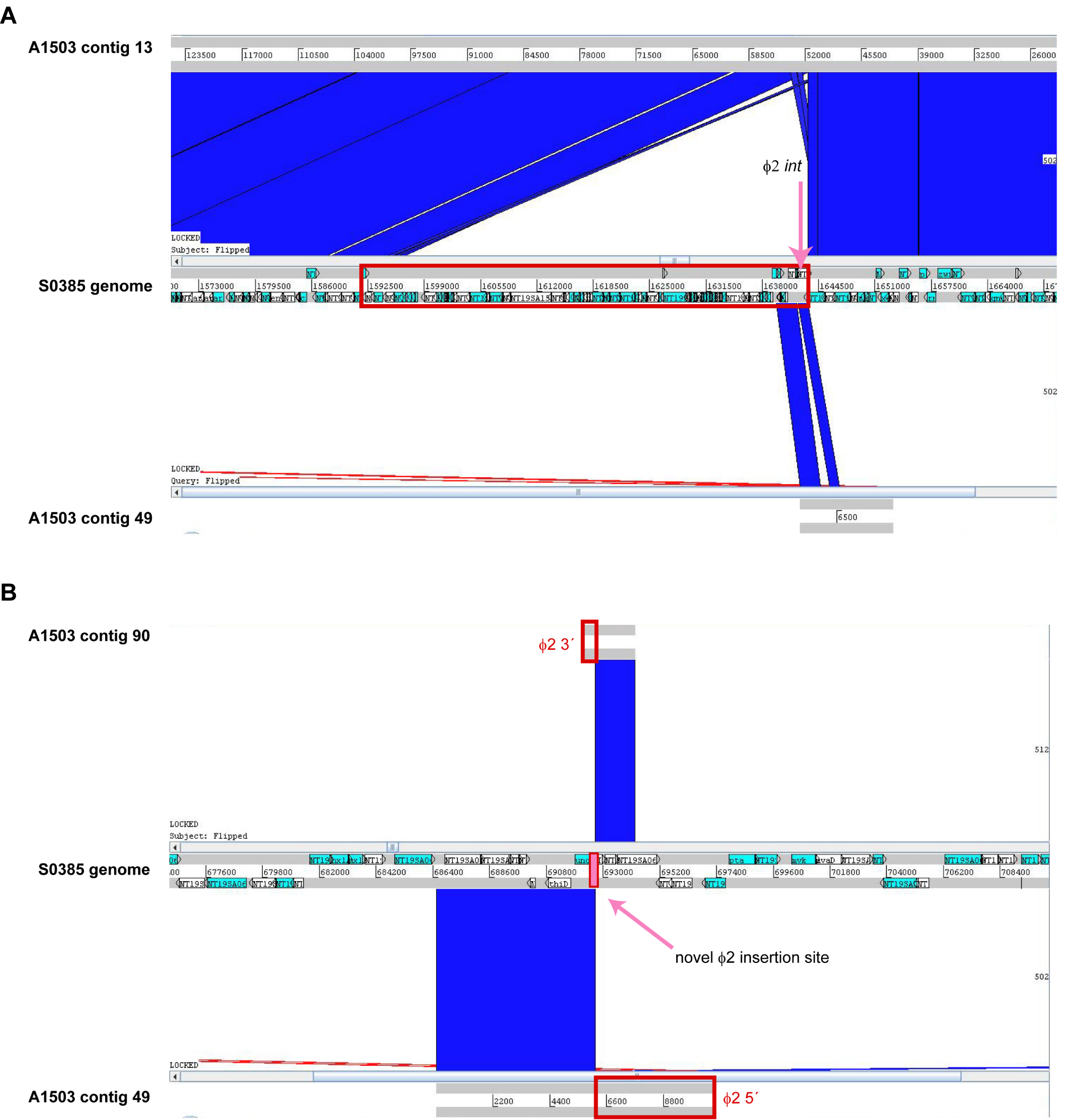
**
